# Human β-defensin-2 production upon viral and bacterial co-infection is attenuated in COPD

**DOI:** 10.1371/journal.pone.0175963

**Published:** 2017-05-10

**Authors:** Jason W. Arnason, James C. Murphy, Cora Kooi, Shahina Wiehler, Suzanne L. Traves, Christopher Shelfoon, Barbara Maciejewski, Curtis J. Dumonceaux, W. Shawn Lewenza, David Proud, Richard Leigh

**Affiliations:** 1 Department of Medicine, Snyder Institute for Chronic Diseases, University of Calgary Cumming School of Medicine, Calgary, Alberta, Canada; 2 Department of Physiology & Pharmacology, Snyder Institute for Chronic Diseases, University of Calgary Cumming School of Medicine, Calgary, Alberta, Canada; 3 Department of Microbiology, Immunology and Infectious Diseases, Snyder Institute for Chronic Diseases, University of Calgary Cumming School of Medicine, Calgary, Alberta, Canada; University of Alabama at Birmingham, UNITED STATES

## Abstract

Viral-bacterial co-infections are associated with severe exacerbations of COPD. Epithelial antimicrobial peptides, including human β-defensin-2 (HBD-2), are integral to innate host defenses. In this study, we examined how co-infection of airway epithelial cells with rhinovirus and *Pseudomonas aeruginosa* modulates HBD-2 expression, and whether these responses are attenuated by cigarette smoke and in epithelial cells obtained by bronchial brushings from smokers with normal lung function or from COPD patients. When human airway epithelial cells from normal lungs were infected with rhinovirus, *Pseudomonas aeruginosa*, or the combination, co-infection with rhinovirus and bacteria resulted in synergistic induction of HBD-2 (p<0.05). The combination of virus and flagellin replicated this synergistic increase (p<0.05), and synergy was not seen using a flagella-deficient mutant *Pseudomonas* (p<0.05). The effects of *Pseudomonas aeruginosa* were mediated via interactions of flagellin with TLR5. The effects of HRV-16 depended upon viral replication but did not appear to be mediated via the intracellular RNA helicases, retinoic acid-inducible gene-I or melanoma differentiation-associated gene-5. Cigarette smoke extract significantly decreased HBD-2 production in response to co-infection. Attenuated production was also observed following co-infection of cells obtained from healthy smokers or COPD patients compared to healthy controls (p<0.05). We conclude that co-exposure to HRV-16 and *Pseudomonas aeruginosa* induces synergistic production of HBD-2 from epithelial cells and that this synergistic induction of HBD-2 is reduced in COPD patients. This may contribute to the more severe exacerbations these patients experience in response to viral-bacterial co-infections.

## Introduction

Chronic Obstructive Pulmonary Disease (COPD) is a leading cause of death worldwide, and In the United States COPD is responsible for about 1 million hospitalizations each year [1, 2]. Acute exacerbations of COPD (AECOPD) account for 3% of all hospitalizations in Canada [3], and for 70% of the health care costs associated with the disease [4, 5]. Current treatment strategies for AECOPD are associated with a substantial failure rate, and patients who suffer from recurrent exacerbations have a worse overall health status, accelerated decline in lung function, increased risk for hospitalization, and a greater mortality rate than those who remain exacerbation free [6, 7]. Respiratory infections are the major trigger of AECOPD [8, 9], with viral infections, particularly human rhinovirus (HRV), being major contributors [10]. Our understanding of the pathogenesis underlying COPD exacerbations is further complicated by the fact that the majority of patients with COPD have chronic bacterial colonization of their lower airways, almost certainly further contributing to COPD exacerbations. Studies have established that approximately 50% of exacerbations are associated with bacterial infections [11]. Furthermore, COPD patients with known bacterial colonization of the airways tend to have increased frequency of exacerbations, as well as a faster decline in lung function compared to patients in which no bacterial infection is detected [12, 13]. Bacteria associated with acute exacerbations include *Steptococcus pneumoniae*, *Haemophilus influenzae*, *Moraxella catarrhalis* and *Pseudomonas aeruginosa* (PA) [14, 15], with PA detected in 5–15% of acute exacerbations in patients with more severe COPD [11]. Thus, viral-bacterial co-infections play a crucial role in the etiology of COPD exacerbations. As such, a number of studies have shown that AECOPD associated with viral-bacterial co-infections are more severe, and are associated with increased length of hospital stay and increased rates of readmission to the hospital compared to exacerbations caused by either viral or bacterial infections alone [14, 16]. The cellular mechanisms by which co-infections lead to worse clinical outcomes in patients with COPD remain poorly understood.

One important variable that may determine susceptibility to AECOPD is variation in the host antimicrobial response. Human β-defensin (HBD)-2 is an antimicrobial peptide (AMP) that plays an integral role in innate immune defenses to infection [17], and studies have shown that either HRV or bacterial infections alone can induce HBD-2 production from the airway epithelium [18–20]. Interestingly, PA induced HBD-2 expression from the airways is reported to be suppressed by cigarette smoke [21], thereby further linking AMP expression to susceptibility of COPD patients to infections. In this study, we tested the hypothesis that co-infection with HRV and PA modulates airway epithelial cell HBD-2 production, and that this response is then dysregulated in epithelial cells obtained from patients with well characterized COPD.

## Materials and methods

All clinical investigation studies were conducted according to the principles expressed in the Declaration of Helsinki. The University of Calgary Conjoint Health Ethics Board approved all protocols for obtaining human cells. Written informed consent was obtained from all subjects prior to bronchoscopy to obtain cells via bronchial brushings. For cells obtained from non-transplanted human lungs, written informed consent was obtained from next of kin at each local site.

### Materials

The following reagents were purchased from the indicated suppliers: bronchial epithelial cell basal medium (BEBM) and additives to create serum-free bronchial epithelial cell growth medium (BEGM) (Lonza, Walkersville, MD); HRV-16 and WI-38 human fetal lung fibroblasts, American Type Tissue Collection (Manassas, VA); HBSS, TRIzol reagent, fetal bovine serum, gentamycin, HEPES buffer, and opti-MEM (Invitrogen, Burlington, ON, Canada); DNase I (Ambion, Austin, TX); 20X GAPDH, RNase inhibitor and reverse transcriptase (Applied Biosystems, Foster City, CA); TaqMan master mix (Roche Diagnostics, Laval, Quebec, Canada); Toll-like receptor (TLR) ligands: lipoteichoic acid, lipopolysaccharide, and *Pseudomonas aeruginosa* flagellin (InvivoGen, San Diego, CA); specific antibodies: TLR5 (Santa Cruz Biotechnology, Santa Cruz, CA), retinoic acid inducible gene-I (RIG-I, Cell Signaling Technology, Danvers, MA), melanoma differentiation-associated protein 5 (MDA5, Enzo Life Sciences, Plymouth Meeting, PA). All other chemicals were purchased from Sigma-Aldrich (Oakville, ON, Canada).

### Epithelial cell cultures

Primary human bronchial epithelial (HBE) cells were obtained via protease digestion of non-transplanted normal human lung tissue as described [22]. Primary cells were grown on six-well culture plates in BEGM. Antibiotics were removed 48 h prior, and hydrocortisone was removed 24 h prior to infection. On the day of infection, HBE cells were pre-treated with BEBM for 2 hours; subsequently, basal medium with the addition of 50 mM HEPES buffer was used for all experimental cell exposures

HBE cells were also obtained from bronchial brushings from healthy, non-smoking individuals, current or former smokers with preserved pulmonary function (healthy smokers) and patients with physician-diagnosed COPD (Stage 1 or 2), according to the Global Initiative for Chronic Obstructive Lung Diseases (GOLD) guidelines for diagnosis of COPD [23]. Characteristics of each subject group are shown in **Table 1**. The University of Calgary Conjoint Health Ethics Board approved all protocols for obtaining human cells.

**Table 1:** Clinical characteristics of healthy non-smokers, healthy smokers, and individuals with confirmed COPD that underwent bronchial biopsies to obtain bronchial brushings.

Characteristics	Normal (n = 5)	Healthy Smoker (n = 5)	COPD (n = 5)
Age, years	36 ± 10.5	58.4 ± 5.9	65.5 ± 5.3
Male/Female	4/1	3/2	4/1
Pack-years of Smoking	N/A	50.4 ± 22.5	47.6 ± 25.6
FEV_1_/FVC	0.79 ± 0.04	0.78 ± 0.05	0.59 ± 0.06
FEV_1_% Predicted	102.4 ± 6.1	96.8 ± 7.9	78.0 ± 19.0

Data are expressed as mean ±SD for each group. FEV_1_ forced expiratory volume in 1 second; FVC forced vital capacity, N/A not applicable.

### Virus and cigarette smoke extract (CSE) preparation

HRV-16 was propagated in WI-38 cells and purified by centrifugation through a sucrose layer to remove ribosomes and soluble factors as previously described [24].

CSE was prepared fresh on the day of stimulation. CSE was generated by bubbling the smoke from one research grade cigarette (3R4F, College of Agriculture Reference Cigarette Program, University of Kentucky) into 4 ml of medium over a period of about 5 minutes using a syringe apparatus, as described [25, 26]. The crude CSE was filtered through a 0.22 mm filter and subsequently adjusted to an absorbance of 0.15 at 320 nm. This solution was considered 100% CSE. All epithelial exposures in the current study were done using 50% CSE, a level which we have previously shown to have no effects on epithelial cell viability [26].

### Bacteria

The wild-type *Pseudomonas aeruginosa* strain PAO1 (PA) and the non-flagellated mutant PAO1 (*fliC*), in which the gene encoding the flagellin protein has been interrupted [27], were plated on Lennox broth (LB) agar (Invitrogen, Burlington, ON, Canada) and incubated overnight at 37°C. Liquid LB cultures were grown for 16–18 h of incubation on a rotating platform at 200rpm at 37°C, the stationary phase bacteria were pelleted at 2000g for 10 minutes, and re-suspended in PBS to a concentration standardized to an optical density at 600nm of 0.2 (~1X10^8^ CFU/ml). Serial dilutions were made and bacterial suspensions plated on LB agar plates for direct bacterial counts to confirm CFU. We confirmed that the *fliC* strain had defective swimming motility in 0.4% agar.

### Stimulation of epithelial cells

HBE cells were stimulated with medium, bacteria, flagellin (30 ng/ml), and CSE alone, or in combination with HRV-16 for 48 h at 34°C in 5% CO_2_, depending on the specific experimental parameters being analyzed. Cells were stimulated with 10^5.5^ 50% tissue culture-infective dose (TCID_50_) U/ml (multiplicity of infection of ~1.0) of HRV-16. Bacterial treatment groups were stimulated with 1X10^5^ CFU/ml of bacteria for 2 h with subsequent bactericidal treatment of 5mM gentamycin. This was done to limit bacterial overgrowth and cytotoxicity of the HBE cells.

Epithelial cell viability was assessed using the 3-(4,5-dimethylthiazol-2-yl)-2,5-diphenyltetrazolium bromide (MTT) assay, as previously described [25].

### Measurement of HBD-2, retinoic acid-inducible gene-I (RIG-I), melanoma differentiation-associated gene 5 (MDA5), and toll-like receptor 5 (TLR 5) proteins

HBD-2 protein expression from HBE cells was measured by ELISA, sensitive to 20 pg/ml, as previously described [18]. Protein levels of TLR5, RIG-I and MDA5 in whole cell lysates were assessed using western blotting as previously described [28].

### RNA extraction and real-time RT-PCR

Total cellular RNA from HBE cells was isolated with TRIzol and treated with DNase. HBD-2 mRNA expression was assessed by real time RT-PCR as described previously [18].

### siRNA knockdown of TLR5, RIG-I and MDA5

Sub-confluent HBE cells were transfected with 10nM of specific siRNAs targeting each molecule of interest, or a control, non-targeting siRNA for 24 h at 37°C using Lipofectamine RNAiMAX in BEGM with antibiotics removed. For each molecule of interest, two different siRNAs were tested to confirm selectivity. After transfection, medium was changed and cells were allowed to recover for 24 h in media without antibiotics and hydrocortisone. Cells were then infected with HRV-16, PA, or the combination of the two. Supernatants and whole-cell lysates from HBE cells were subsequently collected 48 h post infection. The specific forward siRNA sequences used were as follows: TLR 5 duplex A, 5’-GAAUAGCCUUUUAUCGUUUUU-3’; TLR 5 duplex B, 5’-CCAUCUGUUUGAACUUAGAUU-3’; RIG-I duplex A, 5’-AAGCUUUACAACCAGAAUUUA-3’; RIG-I duplex B, 5’-UUCUACAGAUUUGCUCUACUA-3’; MDA5 duplex A, 5’ -CAGAACUGACAUAAGAAUCAA- 3’; and MDA5 duplex B, 5’-CAGGUGUAAGAGAGCUACUAA-3’.

### Statistical analysis

Analysis was performed using GraphPad Prism (GraphPad Software, San Diego, CA). Normally distributed data are presented as mean (±SEM) values, and were analysed using either paired t-tests or one-way ANOVA with Bonferroni’s multiple comparison *post hoc* analysis. Data with two independent variables were analysed using two-way ANOVA with Bonferroni’s multiple comparison *post hoc* analysis. To determine whether there was synergy between HRV-16 and bacteria, the sum of HRV-16 alone and bacteria alone was compared with HRV-16 + bacteria. Paired t-tests or Wilcoxon matched-pairs signed-rank tests were used to determine differences. For all statistical tests, a two-tailed *p* value of <0.05 was considered significant.

## Results

### Co-infection with HRV-16 and *Pseudomonas aeruginosa* induces a synergistic increase in HBD-2 production from human bronchial epithelial cells that is dependent on flagellin

**Fig 1:**
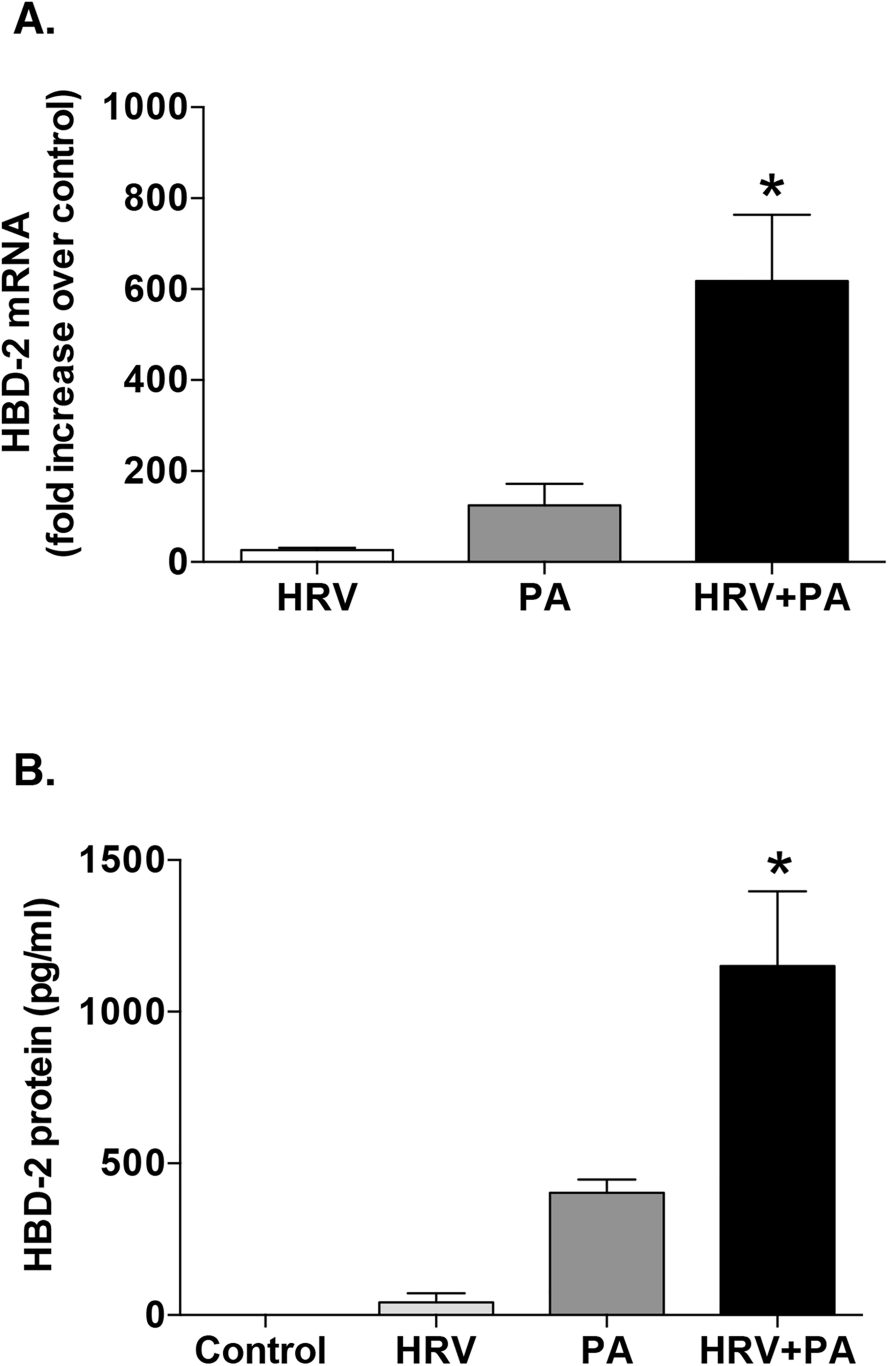
Co-infection of airway epithelial cells with human rhinovirus (HRV) and Pseudomonas aeruginosa (PA) results in a synergistic increase in human beta-defensin (HBD)-2 production. Human bronchial epithelial (HBE) cells were infected with HRV-16 or PA, alone and in combination. HBD-2 mRNA (n = 6) (A) and HBD-2 protein (n = 9) (B) levels were measured 48 h post infection. Data are expressed as mean ±SEM (*p<0.05 between HRV+PA and the sum of the values from each treatment alone).

Based on our prior HRV time-course studies [18], we determined the effect of HRV and PA co-infection on HBD-2 expression from HBE, by measuring levels of HBD-2 mRNA and protein 48 h post infection. Modest HBD-2 mRNA and protein induction was observed following infection with either HRV or PA alone, but the combination of HRV and PA caused a significant, synergistic increase in HBD-2 mRNA expression and in HBD-2 protein production from the HBE cells compared to the other treatment groups (**Fig 1**). Cell viability was confirmed to be >90%, with no overt cytotoxicity 48 h post infection with either HRV or PA, alone and in combination. (See S1 Table for all raw datasets for this paper).

**Fig 2:**
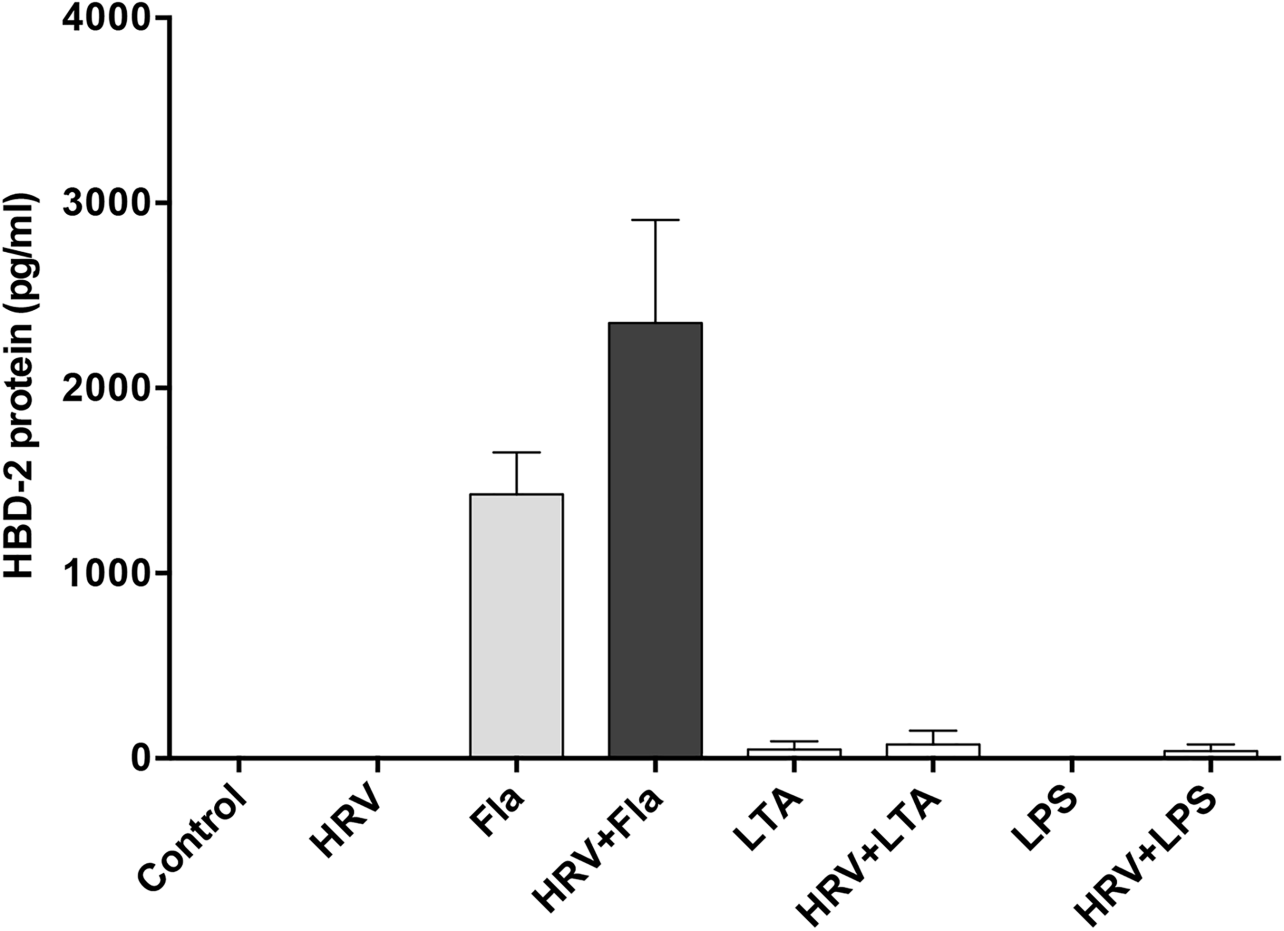
Flagellin (Fla), but not lipoteichoic acid (LTA) or lipopolysaccharide (LPS), induce HBD-2 production from human bronchial epithelial (HBE) cells. HBE cells were treated with medium control, HRV-16 (HRV), Fla (30 ng/mL), LTA (1 μg/mL) LPS (1 μg/mL), alone and in combination with HRV. HBD-2 protein was assessed after 48 h. Data are expressed as mean ±SEM (n = 5).

We evaluated the mechanisms involved in this synergistic HBD-2 response following co-infection with HRV and PA, using known bacterial ligands for toll-like receptors (TLRs), alone and in combination with HRV infection. Stimulation of HBE for 48 h with flagellin (30 ng/ml), a component of bacterial flagella present in PA and a known ligand for TLR5, resulted in a significant upregulation of HBD-2 protein expression. In contrast, lipoteichoic acid (LTA) and lipopolysaccharide (LPS), ligands for TLR2 and TLR4 respectively, even at a concentration of 1 μg/ml, induced little or no HBD-2 production either alone or in combination with HRV infection (**Fig 2**).

**Fig 3:**
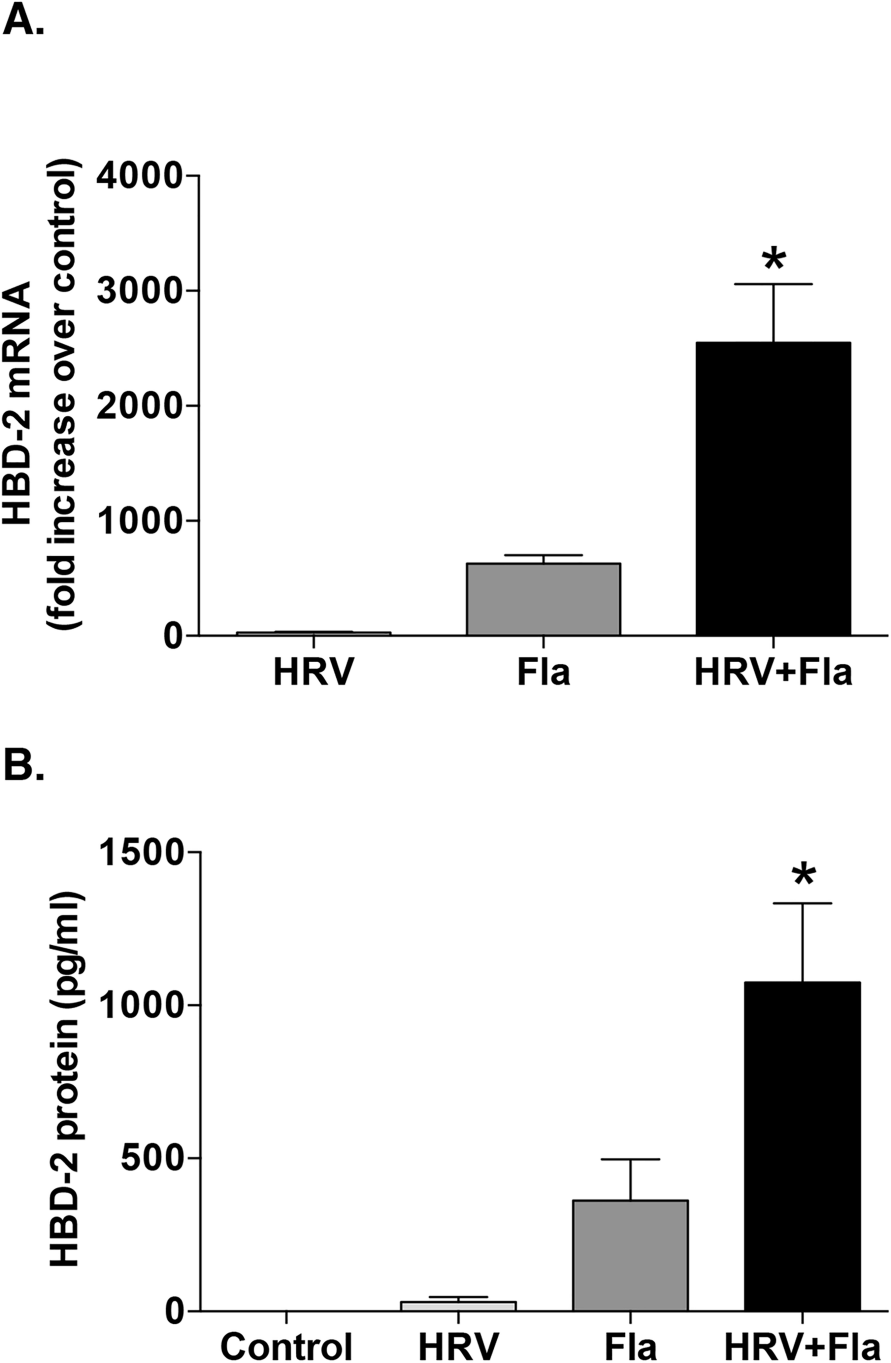
Synergy in human beta-defensin (HBD)-2 expression due to the combination human rhinovirus (HRV)-16 and flagellin (Fla) stimulation mimics HRV and live Pseudomonas aeruginosa (PA) co-infection in airway epithelial cells. **A)** HBD-2 mRNA (n = 8) and **B)** HBD-2 protein (n = 11) levels were determined after stimulation with HRV-16, 30 ng/ml of Fla, or the combination for 48 h in human bronchial epithelial (HBE) cells. Data are expressed as mean ± SEM (*p<0.05 between HRV+Fla and the sum of the values from each treatment alone).

Given that flagellin, but not LTA or LPS, elicited HBD-2 production from HBE cells, we performed additional experiments confirming that flagellin combined with HRV infection synergistically increased HBD-2 mRNA and protein production compared to either HRV or the flagellin protein alone (**Fig 3**). These data suggest that the ability of PA to cause synergistic induction of HBD2 in combination with HRV could be mediated, at least in part, by the flagellin protein present on PA bacteria.

**Fig 4:**
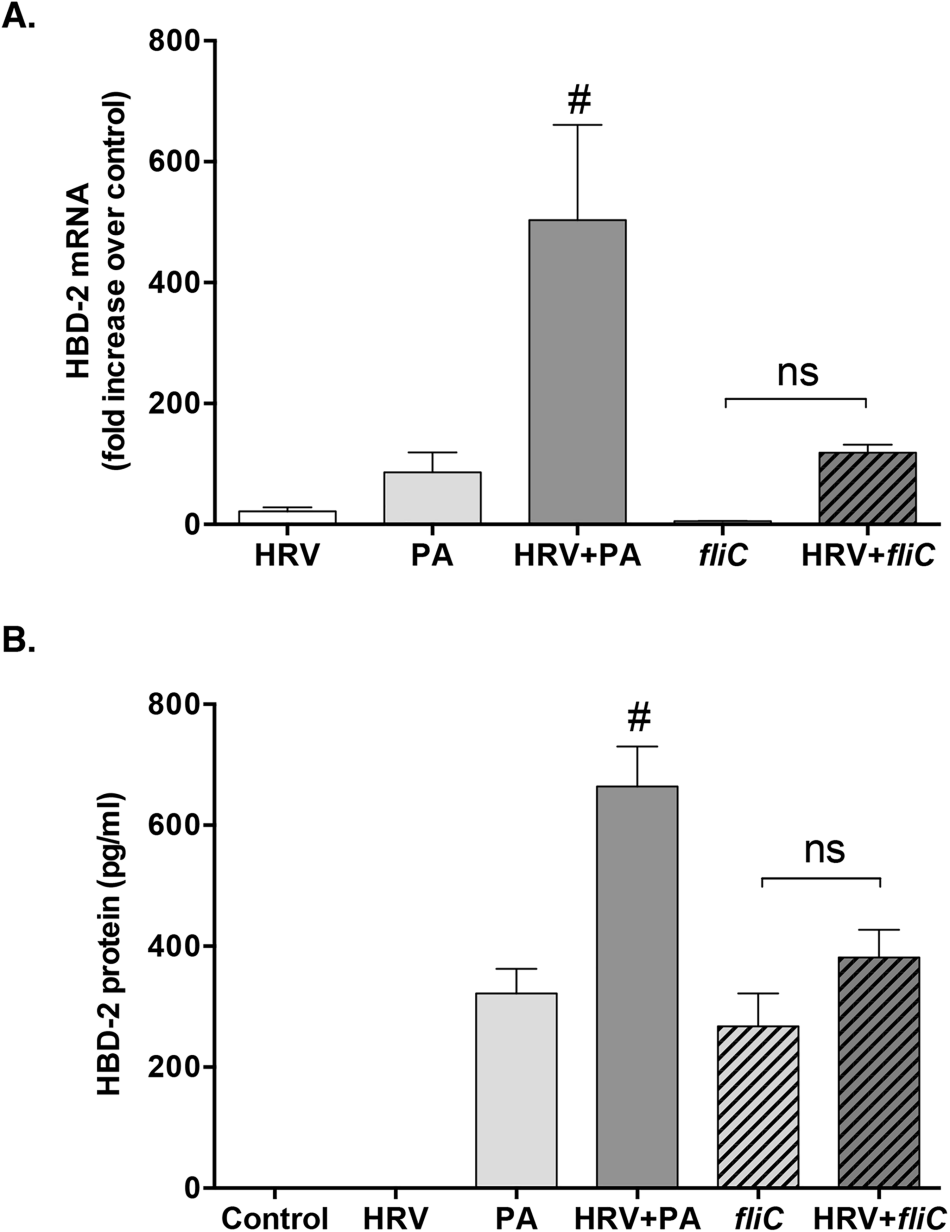
The synergistic response of human beta defensin (HBD)-2 is lost after co-infection with flagellin deficient Pseudomonas aeruginosa (fliC) and human rhinovirus (HRV). HBD-2 mRNA (n = 5) and HBD-2 protein (n = 10) levels were determined following infection of HBE cells for 48 h with HRV-16, wild-type P. aeruginosa (PA), flagellin-deficient P. aeruginosa (fliC) or the combination of either bacterial strain with HRV. Data are expressed as mean ± SEM. Hashtag indicates significant difference compared to all other treatment groups (p<0.05). (ns = not significant).

To further investigate this possibility, we used a mutant strain of *P*. *aeruginosa* (*fliC*), in which the gene encoding for flagellin expression is disrupted, rendering the bacteria flagellin deficient. This *fliC* strain alone was able to induce a modest response of HBD-2 mRNA and protein, similar to the wild-type PA bacterial infection (**Fig 4**). However, synergistic induction of HBD-2 was lost upon co-infection with the *fliC* strain of PA and HRV (**Fig 4**), confirming a role for bacterial flagellin in the synergistic upregulation of HBD-2 protein following HRV and PA co-infection.

### TLR5 knockdown significantly attenuates the synergistic increase in HBD-2 protein that is seen following HRV and *Pseudomonas aeruginosa* co-infection

**Fig 5:**
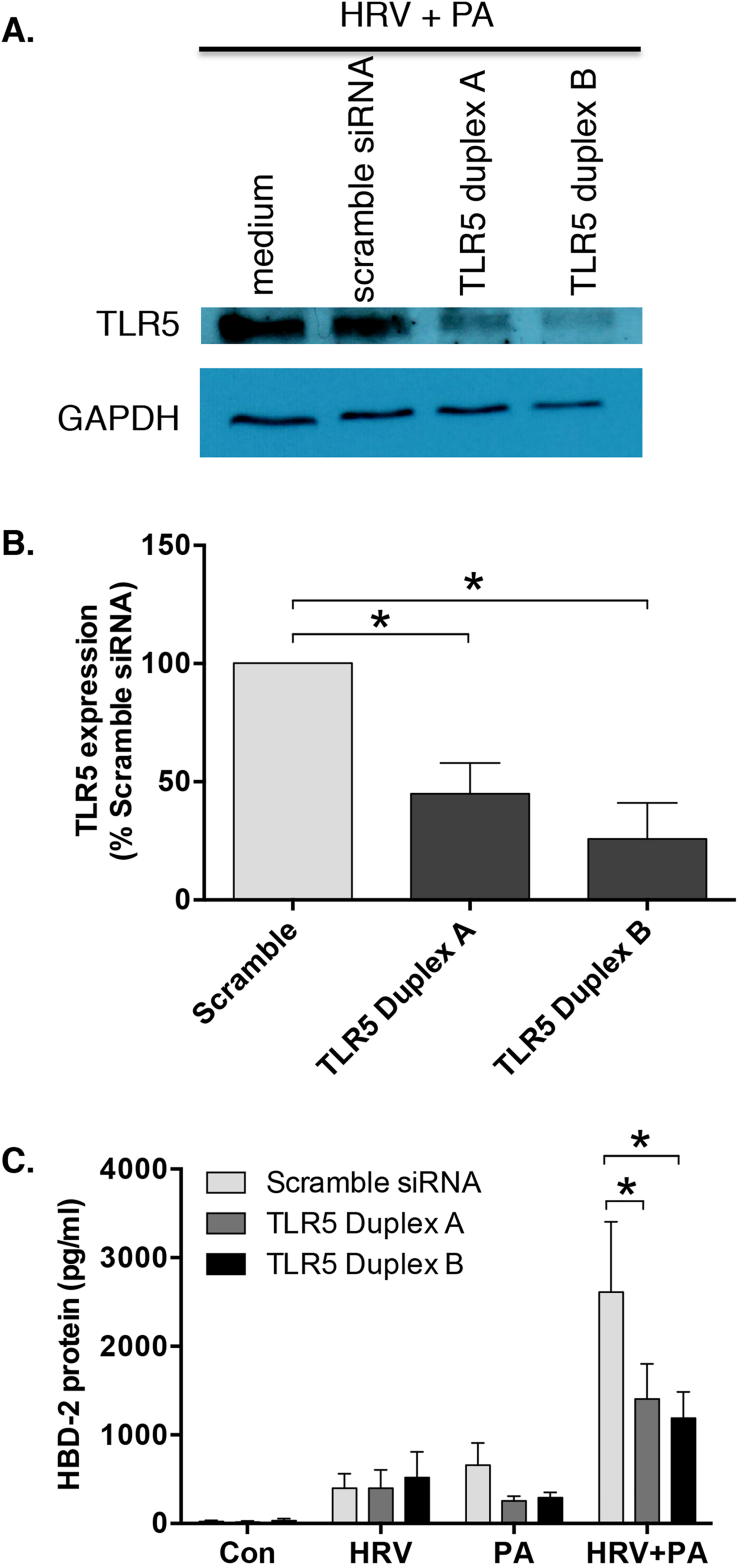
Human rhinovirus (HRV) and Pseudomonas aeruginosa (PA) induced human beta-defensin (HBD)-2 expression is reduced following the knockdown of toll-like receptor (TLR)-5. **A)** TLR5 protein levels in human bronchial epithelial (HBE) cell lysates (48 h after infection) induced following HRV/PA co-infection were assessed via western blotting following lipid-mediated transfection (24 h) of 10 nM TLR5 short-interfering RNA (siRNA) duplexes A or B, compared with non-targeting control siRNA. **B)** Densitometric analysis of TLR5 protein expression of HRV and PA co-infected HBE cells transfected with TLR5 siRNA duplex A or B, or control siRNA. TLR5 expression levels were normalized to GAPDH and compared to siRNA control. **C)** HBD-2 protein (n = 6) levels were determined following 48 h infection with HRV-16, PA, alone and in combination, all in the presence or absence of TLR5 siRNA duplex A or B. Data are expressed as mean ± SEM (*p<0.05).

Given the role of bacterial flagellin in synergistic HBD-2 expression, we next knocked down expression of TLR5 using two different siRNA sequences (designated duplex A and duplex B). Neither the lipid transfection reagent, nor the non-targeting control siRNA altered TLR5 protein levels compared to medium alone (data not shown). Transfection with TLR5 duplex A or duplex B siRNA attenuated TLR5 protein levels in HBE cells, following infection with HRV or PA, alone or in combination, compared to the control siRNA (**Fig 5A**). Densitometric analysis of the western blots (n = 3) indicated that transfection of HBE cells with either of the two TLR5 siRNA duplexes significantly attenuated HBD-2 protein induction in response to co-infection with HRV and PA (**Fig 5B**). Taken together, these data indicate that bacterial flagellin, acting via TLR5, plays a role in mediating the observed synergistic increase in HBD-2 production following HRV and PA co-infection.

### siRNA knockdown of RIG-I and MDA5 does not affect HBD-2 expression in HBE cells

**Fig 6:**
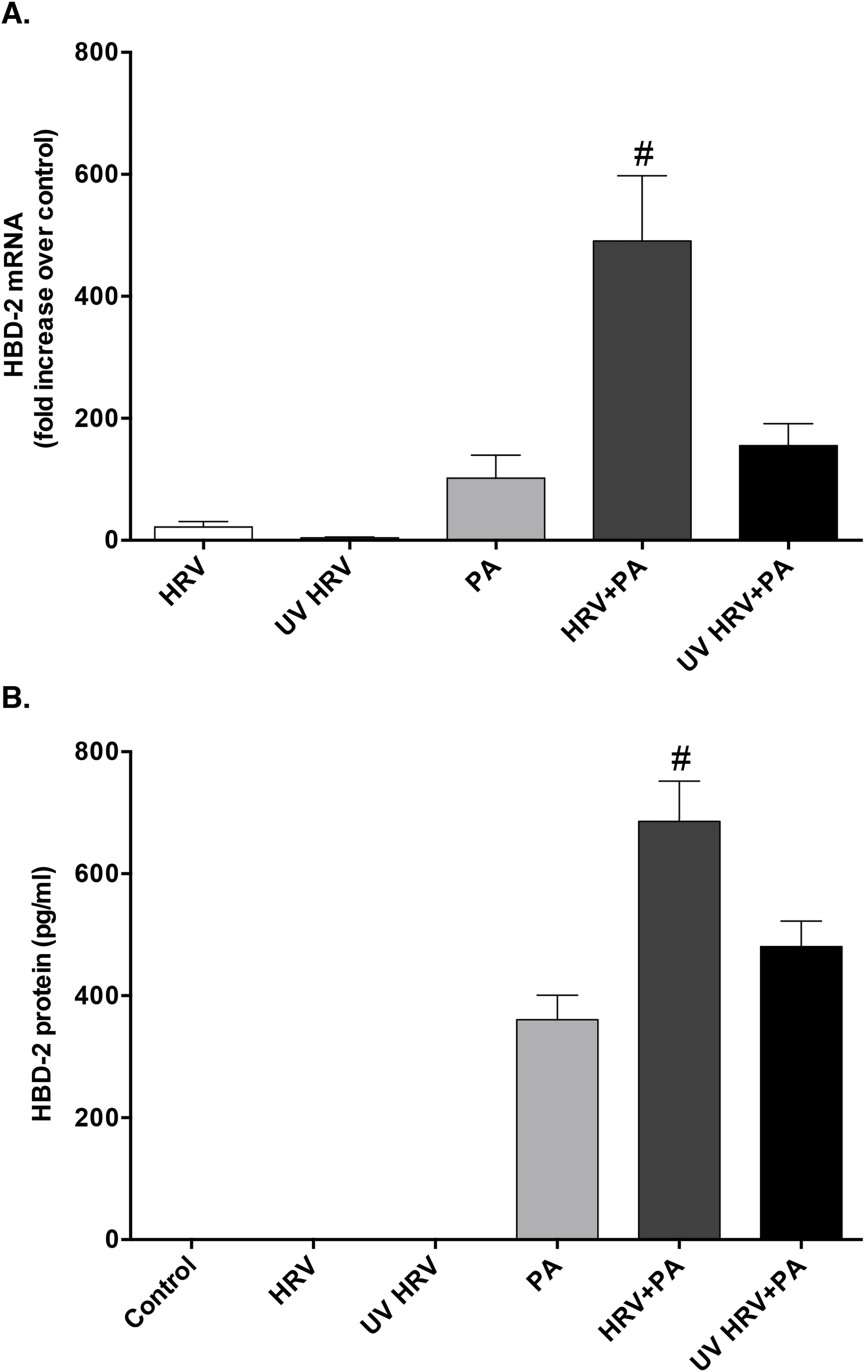
Synergistic expression of HBD-2 is lost after co-infection with replication deficient UV-treated human rhinovirus (UV HRV) and P. aeruginosa (PA). **A)** HBD-2 mRNA (n = 4) and **B)** protein (n = 8) expression from HBE cells after 48 h infection with HRV, PA, UV-HRV, or the combination of PA with either virus group. Data are expressed as mean ± SEM (#p<0.05 compared to all other treatment.

To evaluate whether HRV replication within HBE cells was necessary to drive the synergistic production of HBD-2 protein, HRV was rendered replication deficient by treatment with high-intensity UV-light (UV-HRV). Exposure of HBE cells to UV-HRV, alone and in combination with PA, resulted in significantly lower induction of HBD-2 mRNA and protein levels compared to co-infection with replication intact HRV and PA (**Fig 6**), indicating that intracellular viral replication is required for this response.

**Fig 7:**
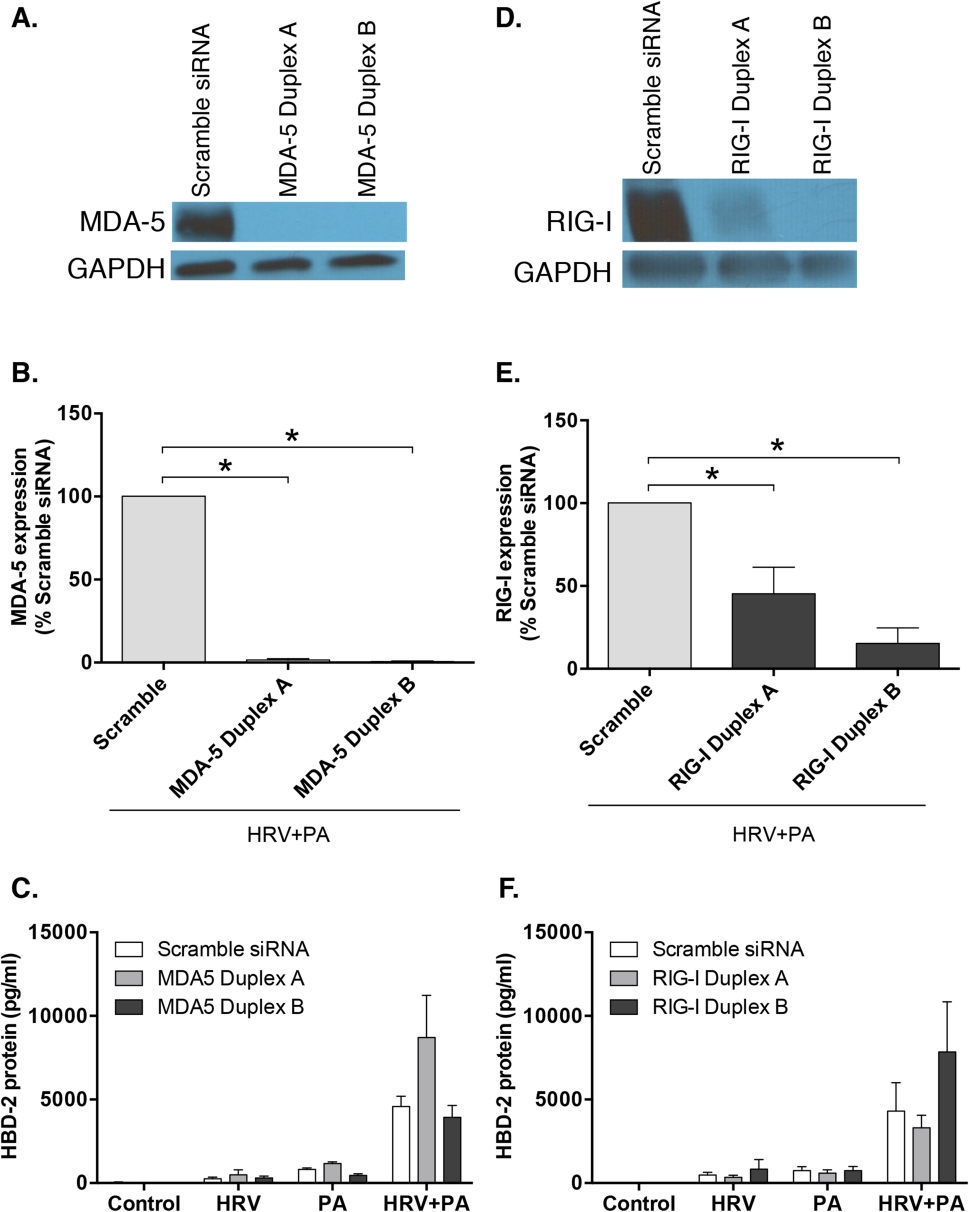
Limited reduction in human beta-defensin (HBD)-2 protein is seen following knockdown of retinoic-acid inducible gene (RIG)-I or melanoma differentiation associated gene-5 (MDA5). **A)** MDA5 and **D)** RIG-I knockdown was assessed via western blot analysis of HBE cells co-infected with human rhinovirus (HRV)-16 and P. aeruginosa (PA) following lipid-mediated transfection (24 h) of 10 nM short-interfering RNA (siRNA) duplex A or B specific for MDA5, compared with non-targeting siRNA control. Densitometric analysis of **B)** MDA5 and **E)** RIG-I knockdown. HBD-2 protein levels were determined 48 h post infection with HRV-16, PA or the combination in the presence or absence of **C)** MDA5 siRNA duplex A or B (n = 4) or **F**) RIG-I siRNA duplex A or B (n = 3). Data expressed as mean ± SEM.

A key intermediate in the replication cycle of HRV is the generation of double-stranded RNA (dsRNA), which can be recognized via pattern recognition receptors, including RIG-I and MDA5 [29]. To examine the role of RIG-I and MDA5 in synergistic induction of HBD-2 expression following co-infection with HRV and PA, siRNA knockdown was used. Despite effective knockdown of MDA5 by both siRNA duplex A and B (**Fig 7A and 7B**), no significant reduction in HBD-2 protein expression was seen, compared to non-targeting control siRNA, in response to co-infection with HRV and PA (**Fig 7C**). Similarly, effective siRNA induced knockdown of RIG-I (**Fig 7D and 7E**) did not attenuate HBD-2 protein release from HBE cells following co-infection with HRV and PA (**Fig 7F**). Thus, neither RIG-I nor MDA5 play a significant role in the synergistic increase in HBD-2 protein production in HBE cells following co-infection with HRV and PA.

### HBD-2 production is significantly attenuated in healthy smokers and COPD patients following HRV and PA co-infection

**Fig 8:**
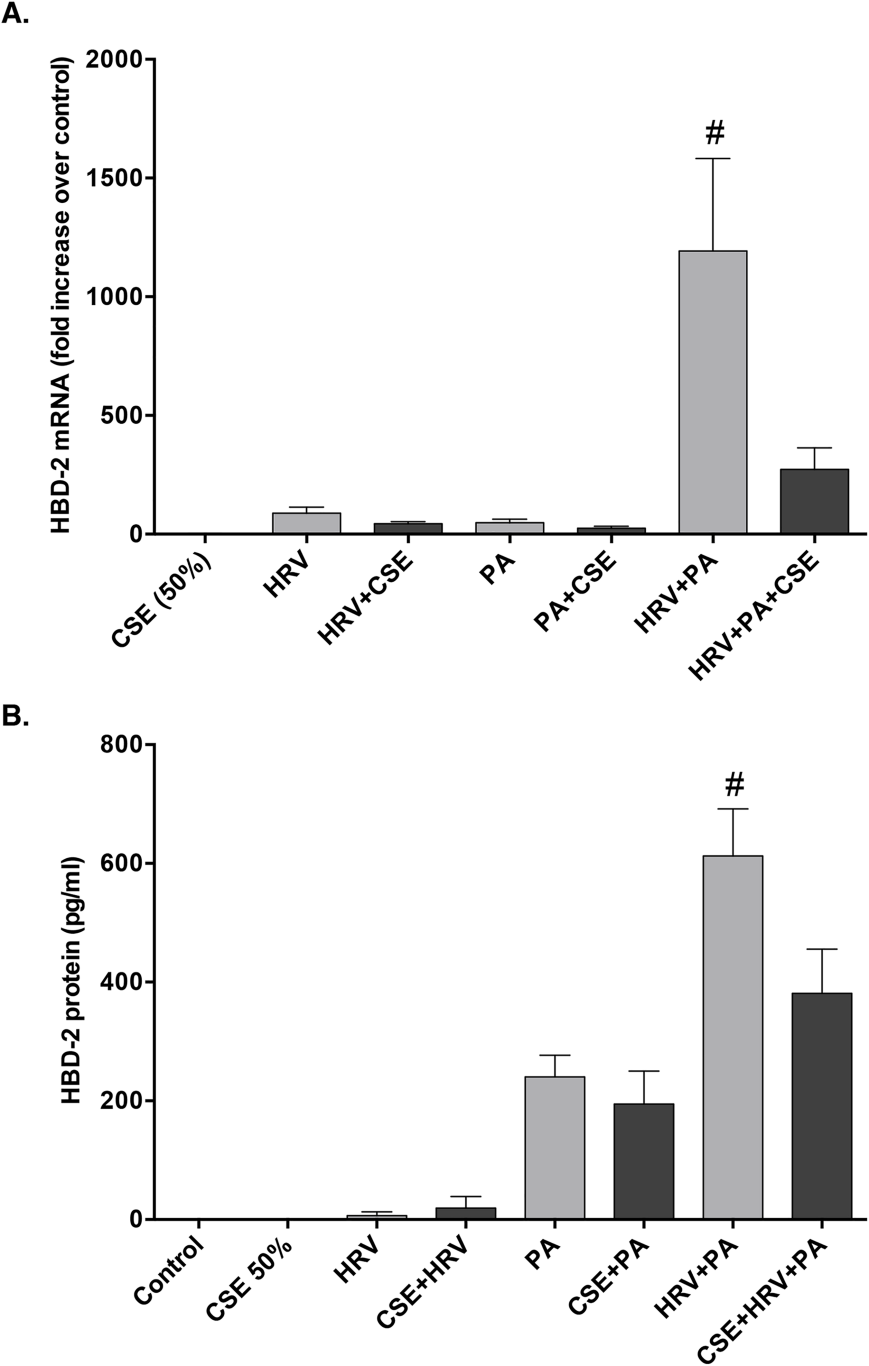
Cigarette smoke extract (CSE) exposure significantly attenuates HBD-2 expression following co-infection with human rhinovirus (HRV)-16 and P. aeruginosa (PA). **A)** HBD-2 mRNA (n = 6) and **B)** protein (n = 8) expression from human bronchial epithelial (HBE) cells following 48 h stimulation with HRV-16, PA, or the combination, with and without addition of 50% CSE. Data expressed as mean ± SEM (#p<0.05 compared to all other treatment groups).

It has been reported that cigarette smoke exposure suppresses the induction of HBD-2 from airway epithelial cells following PA infection [21]. Therefore, we investigated the effects of acute CSE exposure on HBD-2 generation from HBE cells in response to HRV and PA co-infection. Exposure to CSE caused a significant reduction in epithelial HBD-2 mRNA and protein expression following co-infection with HRV and PA (**Fig 8**). Cell viability was confirmed to be >90%, following infection with HRV and PA, and exposure to CSE.

**Fig 9:**
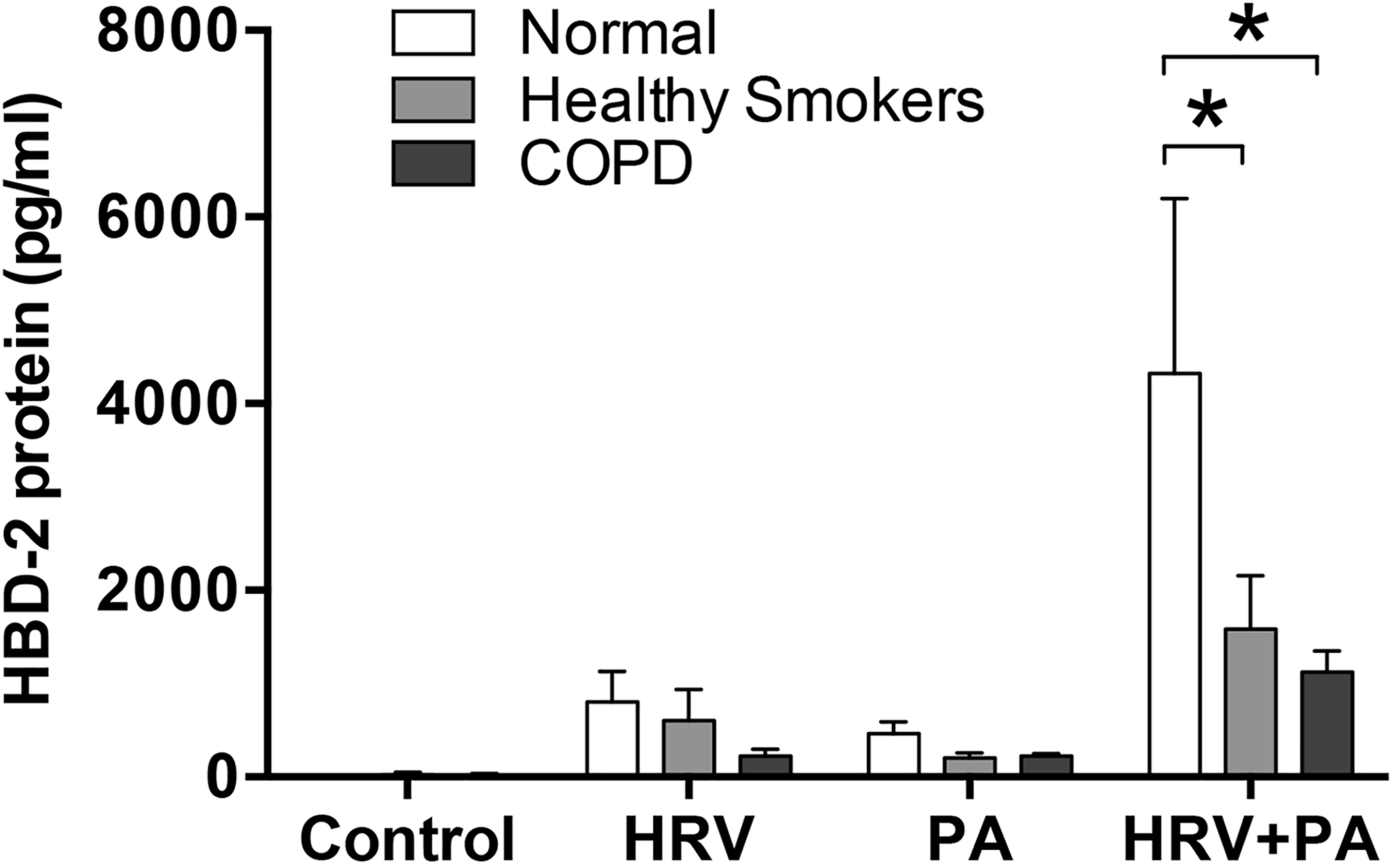
Human beta-defensin (HBD)-2 expression following human rhinovirus (HRV)-16 and P. aeruginosa (PA) co-infection is significantly attenuated in healthy smokers and patients with COPD compared to normal non-smokers. HBD-2 protein expression from epithelial cells obtained during bronchial biopsies of healthy non-smokers (n = 5), healthy smokers with normal lung function (n = 5), and patients with confirmed smoking-related COPD (n = 5) was measured following 48 h infection with HRV-16, PA, or the combination. Data expressed as mean ± SEM (*p<0.05).

Recognizing that acute exposure to CSE may not reproduce responses to chronic smoking, we extended our studies using HBE cells obtained via bronchial brushings from five healthy, non-smoking individuals with normal lung function (controls), five current or former smokers with preserved pulmonary function (post bronchodilator spirometry FEV_1_/FVC ≥0.70 and an FVC above the lower limit of the normal; healthy smokers), and 5 subjects with physician-confirmed, mild to moderate (GOLD stage 1 or 2) smoking-related COPD (**Table 1**). Following HRV and PA co-infection, HBD-2 protein expression from healthy, non-smoking control subjects showed significant synergistic increases in HBD-2 protein expression, similar to the pattern seen in our earlier experiments (**Fig 9**). However, following co-infection with HRV and PA, HBD-2 protein levels were significantly attenuated in HBE cells from both healthy smokers and COPD patients compared to HBD-2 levels measured in HBE cells from healthy controls (**Fig 9**). HBD-2 mRNA production was also attenuated in the healthy smoker and COPD cohorts but, unlike the protein levels where these reductions were significant, the attenuations in mRNA were not statistically significant. This is likely due to inter-subject variability and the small sample size (n = 5) for each of these two cohorts.

## Discussion

In this mechanistic study, we observed a marked synergistic increase in HBD-2 production from airway epithelial cells following co-infection with HRV and PA. While previous studies have reported that infection with either PA or HRV individually can upregulate HBD-2 expression [18, 30, 31] this is, to our knowledge, the first report that viral-bacterial co-infection results in a *synergistic* enhancement of HBD-2 from the airway epithelium. This novel observation implies cross-talk between virally-mediated and bacterially-mediated host defense mechanisms that result in HBD-2 synergy.

To further investigate how the synergistic induction of HBD-2 is mediated following co-infection, we examined cellular components conserved in many bacteria that could drive host immune responses. We exposed HBE cells to conserved components of bacteria, namely LPS, LTA, and flagellin to determine if these factors may play a role in mediating HBD-2 expression to viral-bacterial co-infection. Interestingly, only flagellin was able to upregulate HBD-2 protein expression from HBE cells, whereas neither LPS nor LTA resulted in any appreciable response. Furthermore, the synergy observed following co-infection with PA and HRV was replicated via stimulation with flagellin protein, suggesting that the observed synergistic HBD-2 response noted in HBE cells following HRV and PA co-infection is at least in part due to the flagellin protein expressed on PA.

To confirm that flagellin from PA plays a role in the synergistic induction of HBD-2, we used a mutant strain of PA that lacks flagellin protein expression, and observed a loss of HBD-2 synergy following co-infection with HRV and PA. We, therefore, concluded that the synergistic HBD-2 response to HRV and *P*. *aeruginosa* co-infection is mediated, at least in part, by the flagellin protein found on PA. However, we also observed that HBD-2 protein was still induced following infection with the FliC mutant alone, at levels that were similar to those seen following infection with the wild-type PA. This pointed to other variables that are independent of the extracellular flagellin-mediated signalling, potentially contributing to the synergistic upregulation of HBD-2 expression in HBE cells following co-infection with HRV and PA. In this context, the type III secretion system used by PA has a conserved rod component that contains flagellin protein, which can be sensed by the intracellular NOD-like receptor family caspase recruitment domain-containing protein (NLRC)-4, necessary to drive immune responses [32, 33]. This may point to an additional signaling pathway that could play a role in HBD-2 production from the airway epithelium. Since flagellin is a known ligand for the extracellular receptor TLR5 [34], we examined whether TLR5 knockdown would impact HBD-2 protein production following co-infection with HRV and PA. We confirmed that partial (50–70%) but incomplete knockdown of TLR5 expression using siRNA significantly attenuated HBD-2 protein expression following viral-bacterial co-infection, indicating that TLR5 plays an important role in HBD-2 expression from HBE cells.

To understand how HRV may contribute to the synergistic induction of HBD-2, we first determined whether viral replication was required. To do so, we used replication deficient UV-treated HRV (UV-HRV) and confirmed that there was a loss of HBD-2 synergy from HBE cells following co-infection with UV-HRV and PA, indicating that viral replication is required to drive this synergistic upregulation of HBD-2 following co-infection with HRV and PA. During HRV replication a dsRNA intermediate is formed, which is detected by different innate pattern recognition receptors (PRRs) including TLR3, RIG-I and MDA-5, resulting in a downstream cascade of signaling events. There is some controversy as to the relative role of these PRRs in HRV-induced signalling [29, 35, 36], although RIG-I and MDA-5 have been shown to be induced by HRV infection of primary HBE cells [25]. Therefore, we examined the roles of RIG-I and MDA-5 in the observed synergistic upregulation of HBD-2 protein following viral-bacterial co-infection. Despite effective RIG-I and MDA-5 knockdown, we failed to observe any significant attenuation in HBD-2 protein expression from HBE cells, implying that MDA-5 and RIG-I play a limited role in mediating the synergistic HBD-2 response following HRV and PA co-infection. Thus, other virally-mediated pathways must contribute to this response, but these remain to be delineated.

Since cigarette smoking is the major cause of COPD, we examined its role in regulating HBD-2 production in response to viral-bacterial co-infection. Acute exposure to CSE attenuated HBD-2 mRNA and protein expression from HBE cells following co-infection with HRV and PA. Recognizing that such acute exposure may not reflect chronic smoking we sought to extend our data to the clinical setting, by determining whether the synergistic HBD-2 responses to HRV and PA co-infection was modulated in epithelial cells obtained from either smokers with preserved pulmonary function or patients with GOLD stage 1 or 2 smoking-related COPD, compared to healthy non-smoking individuals. Consistent with the acute CSE exposure data, HBE cells obtained from healthy smokers or patients with COPD both expressed significantly less HBD-2 compared to HBE cells from healthy non-smoking control individuals following co-infection with HRV and PA. Our data are consistent with recently published large clinical studies reporting that rates of respiratory exacerbations among smokers with preserved pulmonary function are significantly higher than among controls who never smoked [37–39]. In addition, our data that HBD-2 levels are significantly lower in airway epithelial cells obtained from healthy smokers with preserved pulmonary function following co-infection with HRV and PA provide a valid biological rationale to account for the observed increase in exacerbations among smokers with preserved pulmonary function reported in these studies. Nonetheless, we acknowledge that the data from our clinical cohorts are limited by small sample size and that more studies are needed to determine the mechanisms that result in epithelial cells from healthy smokers and COPD patients expressing less HBD-2 than non-smokers following HRV and PA co-infection. Interestingly, 4 of the 5 subjects in the heathy smoker cohort were current smokers, suggesting that current cigarette smoke exposure may be a potentially important mechanism by which HBD-2 production is attenuated in healthy smokers. In contrast, only 2 of the 5 COPD subjects were current smokers, suggesting that the smoking-induced pathobiology of COPD may, in its own right, result in attenuation of HBD-2 production. Studies showing that airway epithelial expression of TLR5 is downregulated in both healthy smokers and in smoking-related COPD patients [40] support the concept that HBD-2 expression from the airway epithelium is mediated, in part by the TLR5 signaling pathway. Moreover, our data also point to an altered innate antimicrobial response in chronic smokers, and provide a mechanistic link as to why patients with COPD are more susceptible to viral-bacterial co-infections and have worse clinical outcomes compared to infections with virus or bacteria alone.

In conclusion, co-infection of airway epithelial cells by HRV and PA results in synergistic HBD-2 expression compared to either pathogen alone, and this response is markedly attenuated in epithelial cells obtained from smokers with preserved pulmonary function, as well as in patients with mild-moderate smoking-related COPD. Moreover, this study is the first to provide mechanistic insight into how viral-bacterial co-infections alter the innate antimicrobial response and provides evidence of a dysregulation of the host immune response in COPD. As such, our data provide some rationale into why individuals with COPD have more severe exacerbations in response to viral-bacterial co-infections.

## Supporting information

S1 Table Raw dataset for all figures in this manuscript. (XLSX)

## References

[1] Lozano R, Naghavi M, Foreman K, Lim S, Shibuya K, et al Global and regional mortality from 235 causes of death for 20 age groups in 1990 and 2010: a sytematic analysis for the Global Burden of Disease Study 2010. Lancet. 2012;380:2095–128. doi: 10.1016/S0140-6736(12)61728-0 23245604 10.1016/S0140-6736(12)61728-0 PMC10790329

[2] Guarascio AJ, Ray SM, Finch CK, Self TH. The clinical and economic burden of chronic obstructive pulmonary disease in the USA. Clinicoecon Outcomes Res. 2013;17:235–45. 10.2147/CEOR.S34321 PMC3694800 23818799

[3] Gershon AS, Guan J, Victor JC, Goldstein R, To T. Quantifying health services use for chronic obstructive pulmonary disease. Am J Respir Crit Care Med. 2013;187:596–601. doi: 10.1164/rccm.201211-2044OC 23328526 10.1164/rccm.201211-2044OC

[4] Pauwels RA, Buist AS, Calverley PMA, Jenkins CR, Hurd SS. Global strategy for the diagnosis, management, and prevention of chronic obstructive pulmonary disease. NHLBI/WHO global initiative for chronic obstructive lung disease (GOLD) workshop summary. Am J Respir Crit Care Med. 2001;163:1256–76. doi: 10.1164/ajrccm.163.5.2101039 11316667 10.1164/ajrccm.163.5.2101039

[5] Seemungal TAR, Donaldson CG, Paul EA, Bestall JC, Jeffries DJ, et al Effect of exacerbation on quality of life inpatients with chronic obstructive pulmonary disease. Am J Respir Crit Care Med. 1998;157:1418–22. doi: 10.1164/ajrccm.157.5.9709032 9603117 10.1164/ajrccm.157.5.9709032

[6] Donaldson GC, Seemungal TAR, Bhowmik A, Wedzicha JA. The relationship between exacerbation frequency and lung function decline in chronic obstructive pulmonary disease. Thorax. 2002;57:847–52. doi: 10.1136/thorax.57.10.847 12324669 10.1136/thorax.57.10.847 PMC1746193

[7] Connors AFJ, Dawson NV, Thomas C, Harrell FEJ, Desbiens N, et al Outcomes following acute exacerbation of severe chronic obstructive pulmonary disease. The SUPPORT investigators (Study to understand prognoses and preferences for outcomes and risks of treatment). Am J Respir Crit Care Med. 1996;154:959–67. doi: 10.1164/ajrccm.154.4.8887592 8887592 10.1164/ajrccm.154.4.8887592

[8] Wedzicha JA, Seemungal TAR. COPD exacerbations: defining their cause and prevention. Lancet. 2007;370:786–96. doi: 10.1016/S0140-6736(07)61382-8 17765528 10.1016/S0140-6736(07)61382-8 PMC7134993

[9] Celli BR, Barnes PJ. Exacerbations of chronic obstructive pulmonary disease. Eur Respir J. 2007;29:1224–38. doi: 10.1183/09031936.00109906 17540785 10.1183/09031936.00109906

[10] Seemungal T, Harper-Owen R, Bhowmik A, Moric I, Sanderson G, et al Respiratory viruses, symptoms and inflammatory markers in acute exacerbations and stable chronic obstructive pulmonary disease. Am J Respir Crit Care Med. 2001;164:1618–23. doi: 10.1164/ajrccm.164.9.2105011 11719299 10.1164/ajrccm.164.9.2105011

[11] Sethi S, Murphy TF. Infection in the pathogenesis and course of chronic obstructive pulmonary disease. N Engl J Med. 2008;359:2355–65. doi: 10.1056/NEJMra0800353 19038881 10.1056/NEJMra0800353

[12] Wilkinson TM, Patel IS, Wilks M, Donaldson GC, Wedzicha JA. Airway bacterial load and FEV_1_ decline inpatients with chronic obstructive pulmonary disease. Am J Respir Crit Care Med. 2003;167:1090–5. doi: 10.1164/rccm.200210-1179OC 12684248 10.1164/rccm.200210-1179OC

[13] Patel IS, Seemungal TAR, Wilks M, Lloyd-Owen SJ, Donaldson GC, et al Relationship between bacterial colonization and the frequency, character and severity of COPD exacerbations. Thorax. 2002;57:759–64. doi: 10.1136/thorax.57.9.759 12200518 10.1136/thorax.57.9.759 PMC1746426

[14] Wark PAB, Tooze M, Powell H, Parsons K. Viral and bacterial infection in acute asthma and chronic obstructive pulmonary disease increases the risk of readmission. Respirology. 2013;18:996–1002. doi: 10.1111/resp.12099 23600594 10.1111/resp.12099 PMC7169161

[15] Erb-Downward JR, Thompson DL, Han MK, Freeman CM, McCloskey L, et al Analysis of the lung microbiome in the "healthy" smoker and in COPD. PLoS One. 2011;6:e16384 doi: 10.1371/journal.pone.0016384 21364979 10.1371/journal.pone.0016384 PMC3043049

[16] Wilkinson TMA, Hurst JR, Perera WR, Wilks M, Donaldson GC, et al Effects of interactions between lower airway bacterial and rhinoviral infection in exacerbations of COPD. Chest. 2006;129:317–24. doi: 10.1378/chest.129.2.317 16478847 10.1378/chest.129.2.317 PMC7094441

[17] Jarczak J, Kosciuczuk EM, Lisowski P, Strzalkowska N, Jozwik A, et al Defensins: natural component of human innate immunity. Hum Immunol. 2013;74:1069–79. doi: 10.1016/j.humimm.2013.05.008 23756165 10.1016/j.humimm.2013.05.008

[18] Proud D, Sanders SP, Wiehler S. Human rhinovirus infection induces airway epithelial cell production of human β-defensin-2 both in vitro and in vivo. J Immunol. 2004;172:4637–45. 15034083 10.4049/jimmunol.172.7.4637

[19] Schlee M, Wehkamp J, Altenhoefer A, Oelschlaeger TA, Stange EF, et al Induction of human beta-defensin 2 by the probiotic Escherichia coli Nissle 1917 is mediated through flagellin. Infect Immun. 2007;75:2399–407. doi: 10.1128/IAI.01563-06 17283097 10.1128/IAI.01563-06 PMC1865783

[20] Scharf S, Hippenstiel S, Flieger A, Suttorp N, N'Guessan PD. Induction of human β-defensin-2 in pulmonary epithelial cells by Legionella pneumophilia: involvement of TLR2 and TLR5, p38 MAPK, JNK, NF-κB and AP-1. Am J Physiol Lung Cell Mol Biol. 2010;298:L687–L95. 10.1152/ajplung.00365.2009 20154223

[21] Herr C, Beisswenger C, Hess C, Kandler K, Suttorp N, et al Suppression of pulmonary innate host defence in smokers. Thorax. 2009;64:144–9. doi: 10.1136/thx.2008.102681 18852155 10.1136/thx.2008.102681

[22] Churchill L, Chilton FH, Resau JH, Bascom R, Hubbard WC, et al Cyclooxygenase metabolism of endogenous arachidonic acid by cultured human tracheal epithelial cells. Am Rev Respir Dis. 1989;140:449–59. doi: 10.1164/ajrccm/140.2.449 2504090 10.1164/ajrccm/140.2.449

[23] Global Initiative for Chronic Obstructive Lung Disease. Global strategy for the diagnosis, management and prevention of chronic obstructive pulmonary disease. Report. 2016.

[24] Sanders SP, Siekierski ES, Porter JD, Richards SM, Proud D. Nitric oxide inhibits rhinovirus-induced cytokine production and viral replication in a human respiratory epithelial cell line. J Virol. 1998;72:934–42. 9444985 10.1128/jvi.72.2.934-942.1998 PMC124563

[25] Proud D, Hudy MH, Wiehler S, Zaheer RS, Amin MA, et al Cigarette smoke modulates expression of human rhinovirus-induced airway epithelial host defense genes. PLoS One. 2012;7:e40762 doi: 10.1371/journal.pone.0040762 22808255 10.1371/journal.pone.0040762 PMC3395625

[26] Hudy MH, Traves SL, Wiehler S, Proud D. Cigarette smoke modulates rhinovirus-induced airway epithelial chemokine production. Eur Respir J. 2010;35:1256–63. doi: 10.1183/09031936.00128809 19840959 10.1183/09031936.00128809

[27] Lewenza S, Falsafi RK, Winsor G, Gooderham WJ, McPhee JB, et al Construction of a mini-Tn5-luxCDABE mutant library in Pseudomonas aeruginosa PAO1: a tool for identifying differentially regulated genes. Genome Res. 2005;15:583–9. doi: 10.1101/gr.3513905 15805499 10.1101/gr.3513905 PMC1074373

[28] Wiehler S, Proud D. Interleukin-17A modulates human airway epithelial responses to human rhinovirus infection. Am J Physiol Cell Mol Physiol. 2007;293:L505–L15. 10.1152/ajplung.00066.2007 17545490

[29] Slater L, Bartlett NW, Haas JJ, Zhu J, Message SD, et al Co-ordinated role of TLR-3, RIG-I and MDA5 in the innate response to rhinovirus in bronchial epithelium. PLoS Pathog. 2010;6:e1001178 doi: 10.1371/journal.ppat.1001178 21079690 10.1371/journal.ppat.1001178 PMC2973831

[30] Duits LA, Nibbering PH, van Strijen E, Vos JB, Mannesse-Lazeroms SP, et al Rhinovirus increases human β-defensin-2 and -3 mRNA expression in cultured human bronchial epithelial cells. FEMS Immunol Med Microbiol. 2003;38:59–64. 12900056 10.1016/S0928-8244(03)00106-8

[31] Harder J, Meyer-Hoffert U, Teran LM, Schwichtenberg L, Bartels J, et al Mucoid Pseudomonas aeruginosa, TNF-α, and IL-1β, but not IL-6, induce human β-defensin-2 in respiratory epithelia. Am J Respir Cell Mol Biol. 2000;22:714–21. doi: 10.1165/ajrcmb.22.6.4023 10837369 10.1165/ajrcmb.22.6.4023

[32] Zhao Y, Yang J, Shi J, Gong YN, Lu Q, et al The NLRC4 inflammasome receptors for bacterial flagellin and type III secretion apparatus. Nature. 2011;477:596–600. doi: 10.1038/nature10510 21918512 10.1038/nature10510

[33] Miao EA, Mao DP, Yudkovsky N, Bonneau R, Lorang CG, et al Innate immune detection of the type III secretion apparatus through the NLRC4 inflammasome. Proc Natl Acad Sci U S A. 2010;107:3076–80. doi: 10.1073/pnas.0913087107 20133635 10.1073/pnas.0913087107 PMC2840275

[34] Hayashi F, Smith KD, Ozinsky A, Hawn TR, Yi EC, et al The innate immune response to bacterial flagellin is mediated by Toll-like receptor 5. Nature. 2001;410:1099–103. doi: 10.1038/35074106 11323673 10.1038/35074106

[35] Wang Q, Miller DJ, Bowman ER, Nagarkar DR, Schneider D, et al MDA5 and TLR3 initiate pro-inflammatory signaling pathways leading to rhinovirus-induced airways inflammation and hyperresponsiveness. PLoS Pathog. 2011;7:e1002070 doi: 10.1371/journal.ppat.1002070 21637773 10.1371/journal.ppat.1002070 PMC3102730

[36] Triantafilou K, Vakakis E, Richer EAJ, Evans GL, Villiers JP, et al Human rhinovirus recognition in non-immune cells is mediated by Toll-like receptors and MDA-5, which trigger a synergetic pro-inflammatory immune response. Virulence. 2011;2:1–8. 21224721 10.4161/viru.2.1.13807 PMC3073236

[37] Tan WC, Bourbeau J, Hernandez P, Chapman KR, Cowie R, et al Exacerbation-like respiratory symptoms in individuals without chronic obstructive pulmonary disease: results from a population-based study. Thorax. 2014;69:709–17. doi: 10.1136/thoraxjnl-2013-205048 24706040 10.1136/thoraxjnl-2013-205048 PMC4112491

[38] Regan EA, Lynch DA, Curran-Everett D, Curtis JL, Austin JH, et al Clinical and radiological disease in smokers with normal spirometry. JAMA Intern Med. 2015;175:1539–49. doi: 10.1001/jamainternmed.2015.2735 26098755 10.1001/jamainternmed.2015.2735 PMC4564354

[39] Woodruff PG, Barr RG, Bleecker E, Christenson SA, Couper D, et al Clinical significance of symptoms in smokers with preserved pulmonary function. N Engl J Med. 2016;374:1811–21. doi: 10.1056/NEJMoa1505971 27168432 10.1056/NEJMoa1505971 PMC4968204

[40] Wang R, Ahmed J, Wang G, Hassan I, Strulovici-Barel Y, et al Airway epithelial expression of TLR5 is downregulated in healthy smokers and smokers with chronic obstructive pulmonary disease. J Immunol. 2012;189:2217–25. doi: 10.4049/jimmunol.1101895 22855713 10.4049/jimmunol.1101895 PMC3579667

